# Comparison of Masticatory Efficiency Between Single-Piece Implant-Supported Crowns and Conventional Fixed Partial Dentures for Single Posterior Tooth Replacement: A 12-Month Prospective Observational Study

**DOI:** 10.7759/cureus.101229

**Published:** 2026-01-10

**Authors:** Sujeet K Patil, Nagsen P Kamble, Sasankoti Mohan Ravi Prakash, Balaji Dhanraj Kendre, Khalid Mohammed AgwanI, Dev Kumar Garg, Rahul Tiwari, Heena Dixit, Seema Gupta

**Affiliations:** 1 Department of Prosthodontics, Crown and Bridge, Bharati Vidyapeeth (Deemed to be University) Dental College and Hospital, Sangli, IND; 2 Department of Forensic Medicine and Toxicology, Dr. Vithalrao Vikhe Patil Foundation’s Medical College and Hospital, Ahilyanagar, IND; 3 Department of Dentistry, Ozarks Community Health Center, Stockton Dental Clinic, Stockton, USA; 4 Department of Orthodontics, Saraswati Dhanwantari Dental College and Hospital, Parbhani, IND; 5 Department of Oral and Maxillofacial Surgery, Darshan Dental College and Hospital, Udaipur, IND; 6 Department of Oral and Maxillofacial Surgery, Geetanjali Dental and Research Institute, Udaipur, IND; 7 Department of Oral and Maxillofacial Surgery, RKDF Dental College and Research Centre, Bhopal, IND; 8 Department of Blood Cell, Commissionerate of Health and Family Welfare, Hyderabad, IND; 9 Department of Orthodontics, Kothiwal Dental College and Research Centre, Moradabad, IND

**Keywords:** dental implants, fixed partial dentures, masticatory, performance, prospective

## Abstract

Introduction: Single posterior tooth loss impairs masticatory function, dietary choices, and quality of life. This prospective clinical study compared masticatory efficiency and functional adaptation between single-piece implant-supported crowns and conventional tooth-supported fixed partial dentures (FPDs) over a 12-month period.

Materials and methods: Sixty partially edentulous patients (aged 25-60 years) requiring single posterior tooth replacement were consecutively allocated into two equal groups (n = 30 each). Group 1 received single-piece titanium implants with cemented monolithic zirconia crowns after three-month osseointegration. Group 2 received conventional three-unit metal-ceramic FPDs. Masticatory efficiency was assessed at three-, six-, and 12-month post-loading using a standardized almond comminution test. The chewed particles were dried and sieved, and the masticatory performance (MP%) was calculated. Data were analyzed using independent-sample t-tests for intergroup comparisons and repeated-measures analysis of variance (ANOVA) with Bonferroni post-hoc tests for intragroup changes (p < 0.05).

Results: All 60 patients completed the study with comparable baseline characteristics. The implant group showed significantly higher MP than the FPD group at all time points: 28.4 ± 4.2% vs. 22.6 ± 3.8% at three months (p = 0.001), 32.1 ± 3.7% vs. 25.4 ± 3.9% at six months (p = 0.001), and 35.7 ± 3.9% vs. 28.3 ± 4.1% at 12 months (p = 0.001). Both groups improved significantly over time, with Bonferroni tests confirming significant gains between all the intervals in each group. The implant group showed greater improvement throughout the follow-up period.

Conclusion: Single-piece implant-supported crowns provided superior masticatory efficiency and faster functional adaptation than conventional FPDs for single posterior tooth replacement.

## Introduction

Restoration of lost posterior teeth is one of the most common clinical challenges in prosthodontics because masticatory function directly influences dietary choices, nutritional intake, and overall quality of life [1]. Conventional tooth-supported fixed partial dentures (FPDs) have long been the gold standard for replacing single missing teeth; however, they require irreversible preparation of adjacent healthy teeth, which may carry the risk of secondary caries and transmission of occlusal loads through the periodontal ligament (PDL) of abutment teeth, which may limit force generation owing to protective proprioceptive reflexes [2].

The introduction of osseointegrated dental implants has revolutionized tooth replacement by providing independent, bone-anchored support without compromising adjacent teeth [3]. Single-piece implants, with their integrated transgingival abutment-fixture design, further simplify the restorative process by eliminating the microgap at the implant-abutment interface, reducing peri-implant inflammation, and allowing predictable one-stage surgery and early loading protocols [4]. Several studies have reported that implant-supported restorations achieve higher maximum bite forces and better masticatory performance (MP) than conventional bridges, approaching the values of natural dentition [5,6]. These functional advantages are attributed to rigid bone anchorage, the absence of PDL-mediated feedback, and optimal occlusal contact distribution without pontics.

Despite the growing popularity of single-piece implant systems, prospective clinical trials comparing masticatory efficiency and bite force between single-piece implant-supported crowns and conventional three-unit FPDs for single posterior tooth replacement remain scarce. Most existing comparative studies have involved two-piece implants or multiple-unit restorations, limiting their applicability to simplified single-piece protocols. Therefore, the present prospective clinical study aimed to compare masticatory efficiency and maximum bite force in patients restored with single-piece titanium implants versus conventional tooth-supported FPDs for single posterior tooth replacement over a 12-month period. The specific objectives were to quantify and compare masticatory efficiency at three, six, and 12 months post-loading, and to evaluate the progression of functional adaptation in both treatment groups over time. 

## Materials and methods

Study design and setting

This prospective, parallel-group observational study was conducted at the Department of Prosthodontics, Ram Krishna Dharmarth Foundation (RKDF) Dental College and Research Centre, India. The study spanned from March 2021 to December 2023 and encompassed patient recruitment, treatment procedures, and a 12-month follow-up period. The study was approved by the Institutional Ethics Committee prior to initiation and in compliance with the Declaration of Helsinki guidelines for human research. Written informed consent was obtained from all participants after a detailed explanation of the study objectives, procedures, potential risks, and benefits, with participants having the right to withdraw at any stage without prejudice.

Participant eligibility criteria

Sixty partially edentulous patients requiring replacement of a single posterior tooth (premolar or first molar) were recruited based on the stringent inclusion and exclusion criteria. Inclusion criteria were individuals aged 25-60 years, with adequate alveolar bone volume for implant placement (minimum 10 mm height and 6 mm width as assessed via cone-beam computed tomography), healthy abutment teeth for FPDs without caries or periodontal disease, and absence of parafunctional habits such as bruxism. Exclusion criteria were uncontrolled systemic conditions (such as diabetes with HbA1c >7% and cardiovascular diseases), heavy smoking (>10 cigarettes per day), history of head and neck radiotherapy, temporomandibular joint disorders, or any contraindication to surgical procedures. The patients were screened through comprehensive clinical examinations, radiographic evaluations, and medical history reviews to ensure homogeneity and minimize confounding factors.

Sample size estimation

The sample size was determined using the G*Power software (version 3.1.9.2; Heinrich Heine University, Düsseldorf, Germany). Utilizing a clinically applicable effect size (0.64) for the primary outcome of masticatory efficiency from a reference study by Kumar et al. [7], a sample of 30 subjects per group was required to ensure 80% power at 5% (0.05) significance level. Eligible participants were consecutively allocated to two equal groups (n = 30 each). Group 1 received single-piece titanium implants, while Group 2 received conventional three-unit metal-ceramic FPDs. Blinding of participants and operators was not feasible because of the distinct nature of the treatments, but outcome assessors were blinded to group assignments during functional evaluations.

Treatment protocols

For Group 1, single-piece titanium implants (diameter 3.75-4.2 mm, length 10-12 mm; product: Equinox Myriad One-Piece Implant System, Equinox Medical Technologies B.V., Amersfoort, Netherlands) were placed following a two-stage surgical protocol. Initial osteotomy was performed under local anesthesia, with implants inserted at the bone level and covered with healing caps for a three-month osseointegration period before prosthetic loading. The final restorations consisted of monolithic zirconia crowns cemented onto integrated abutments. For Group 2, conventional FPDs were fabricated using metal-ceramic materials (base alloy: Wiron 99, Bego GmbH & Co. KG, Bremen, Germany; porcelain, VMK Master, VITA Zahnfabrik H. Rauter GmbH & Co. KG, Bad Säckingen, Germany). Abutment teeth were prepared conservatively, impressions were taken with polyvinyl siloxane (Aquasil Ultra, Dentsply Sirona, Charlotte, NC, USA), and prostheses were cemented using glass ionomer cement (GC FujiCEM 2, GC Corporation, Tokyo, Japan). All procedures adhered to standardized prosthodontic guidelines, with occlusal adjustments performed to ensure balanced contact.

Assessment of masticatory efficiency

Masticatory efficiency was evaluated using almonds as a natural test food following a standardized protocol adapted from previously validated methods [8,9]. To avoid measurement bias, all assessments were performed by a single trained examiner who was blinded to the type of prosthetic rehabilitation. Each participant received five whole almonds and was instructed to chew them at a comfortable and natural pace for 20 mandibular strokes. The examiner counted the strokes to ensure accuracy and collected fragmented material in a sterile container immediately after chewing. To retrieve any residual particles from the oral cavity, the participant was rinsed with 50 mL of water, and the rinse was added to the same container. The combined sample (chewed almonds + rinse water) was poured onto a stainless steel sieve (dimensions approximately 175 × 78 × 40 mm) lined with filter paper. To remove saliva and prevent the agglomeration of particles, an additional 500 mL of water was passed through the sieve. Only the solid particles retained on the filter were used for subsequent analysis.

The collected almond fragments were transferred to a laboratory drying oven (Fanem, São Paulo, Brazil) and dehydrated at 130°C for 40 min to standardize moisture content. Once dried, the sample was subjected to particle size separation using a graded four-sieve assembly (Granutest, Telastem, São Paulo, Brazil), approved by the Brazilian Association of Technical Standards (ABNT). The sieves had calibrated apertures of 4.0 mm, 2.8 mm, 2.0 mm, and 1.0 mm. The stacked assembly was placed on a gypsum vibrator and shaken for 60 s to achieve uniform distribution of particles across the sieves. After sieving, the contents from each layer and the base collector were weighed using a high-precision digital balance (Gehaka Ltd., São Paulo, Brazil). The total dry mass was used as the reference (Pt). The proportion of finely fragmented particles, defined as the weight passing through the 2.8-mm sieve and smaller, was summed (P1). MP was derived using the formula given by Kapur and Soman [10] (Permission to use the formula has been obtained). Higher MP values indicated more effective comminution of the almonds.

Standardization and examiner reliability

To ensure methodological consistency, the examiner performed calibration using repeated measurements of pilot samples. The protocol yielded a high degree of reproducibility, with intraclass correlation values above the acceptable limits, confirming reliable assessment conditions. Assessments of both masticatory efficiency and bite force were conducted at three, six, and 12-month post-prosthetic loading by trained, blinded evaluators.

Statistical analysis

Data were analyzed using the Statistical Package for Social Sciences (SPSS) version 23.0 (IBM Corp., Armonk, NY, USA). Baseline categorical variables were compared between the groups using the chi-square (χ²) test. The distribution of masticatory performance (MP%) data at each follow-up interval was assessed using the Shapiro-Wilk test. As the outcome data met normality assumptions, parametric tests were applied. Intergroup comparisons of MP% at each time point were performed using independent-sample t-tests. Intragroup changes over time (three, six, and 12 months) were evaluated using repeated-measures analysis of variance (ANOVA) followed by Bonferroni-adjusted post-hoc tests. Statistical significance was set at p <0.05.

## Results

All 60 participants completed the study. At baseline, there were no significant differences between the single-piece implant and FPD groups in terms of age, sex distribution, missing tooth region, duration of edentulism, and baseline MP (all p > 0.05). This confirmed that both groups were comparable before treatment (Table 1).

**Table 1:** Baseline demographic and clinical characteristics of participants (n = 30 per group). Values are presented as mean ± standard deviation (SD) for continuous variables and as frequencies for categorical variables. Independent-sample t-tests (t values) were used for continuous variables, and chi-square (χ²) tests were used for categorical variables. p-Value >0.05 denotes no statistical significance.

Variable		Single-piece implant (n = 30)	Conventional FPD (n = 30)	Test value	p-Value
Sex	Male	17 (56%)	16 (53%)	χ² = 0.07	0.79
	Female	13 (44%)	14 (47%)		
Age (years)	Mean ± SD	44.7 ± 8.3	45.1 ± 7.9	t = –0.23	0.82
Missing tooth	Premolar	12 (40%)	11 (37%)	χ² = 0.08	0.77
	Molar	18 (60%)	19 (63%)		
Duration of edentulism (months)	Mean ± SD	6.8 ± 2.4	6.5 ± 2.1	t = 0.48	0.63
Baseline masticatory performance (MP%) [10]	Mean ± SD	21.8 ± 4.1	21.3 ± 3.8	t = 0.47	0.64

At all follow-up intervals, the single-piece implant group demonstrated a significantly higher MP than the conventional FPD group. At three months, the implant group achieved a mean MP% of 28.4 ± 4.2%, which was significantly greater than the 22.6 ± 3.8% observed in the FPD group (t = 5.60, p = 0.001). This difference widened over time, with mean MP% values of 32.1 ± 3.7% versus 25.4 ± 3.9% at six months (t = 6.82, p = 0.001) and 35.7 ± 3.9% versus 28.3 ± 4.1% at 12 months (t = 7.16, p = 0.001). These findings indicated that implant-supported restorations consistently provided superior comminution efficiency throughout the study period (Table 2).

**Table 2:** Comparison of MP% between groups (independent t-test). Values are presented as mean ± standard deviation (SD); intergroup comparisons at each time point were performed using independent-sample t-tests. *p-Value <0.05 was considered statistically significant. MP% = masticatory performance percentage derived from almond comminution analysis [10].

Time points	Single-piece implants (n = 30)	Conventional FPDs (n = 30)	T stats	p-Value
	Mean ± SD	Mean ± SD		
3 months	28.4 ± 4.2	22.6 ± 3.8	5.60	0.001*
6 months	32.1 ± 3.7	25.4 ± 3.9	6.82	0.001*
12 months	35.7 ± 3.9	28.3 ± 4.1	7.16	0.001*

Both the implant and FPD groups exhibited significant improvements in MP% across the three follow-up intervals. In the implant group, MP% increased from 28.4 ± 4.2% at three months to 32.1 ± 3.7% at six months and 35.7 ± 3.9% at 12 months, with repeated-measures ANOVA confirming a significant time-dependent effect (F = 25.71, p = 0.001). Similarly, the FPD group showed progressive enhancement from 22.6 ± 3.8% to 25.4 ± 3.9% and 28.3 ± 4.1%, also yielding a statistically significant temporal trend (F = 15.73, p = 0.001). However, the higher F-value in the implant group suggests a more robust functional improvement compared with conventional FPDs (Table 3).

**Table 3:** Intragroup comparison of masticatory performance over time (repeated-measures ANOVA). Values are presented as mean ± standard deviation (SD), temporal changes within each group were analyzed using repeated-measures ANOVA, followed by Bonferroni-adjusted post hoc testing when appropriate. *p-Value <0.05 indicates statistically significant change over time. MP% = masticatory performance percentage obtained from the standardized almond fragmentation method [10]; ANOVA = analysis of variance.

Groups	3 months	6 months	12 months	F-value	p-Value
	Mean ± SD	Mean ± SD	Mean ± SD		
Single-piece implants (n = 30)	28.4 ± 4.2	32.1 ± 3.7	35.7 ± 3.9	25.71	0.001*
Conventional FPDs (n = 30)	22.6 ± 3.8	25.4 ± 3.9	28.3 ± 4.1	15.73	0.001*

Bonferroni-adjusted pairwise comparisons revealed significant gains in MP% between all assessed intervals in both treatment groups. In the implant group, improvements were significant between three and six months (t = -4.92, p = 0.001), three and 12 months (t = -5.40, p = 0.001), and six and 12 months (t = -8.16, p = 0.001). The FPD group also demonstrated significant improvements between three and six months (t = -3.88, p = 0.001), three and 12 months (t = -3.74, p = 0.001), and six and 12 months (t = -6.28, p = 0.001). Although both groups showed gradual improvement, the magnitude of the change was consistently larger in the implant group, indicating greater functional adaptation over time (Table 4).

**Table 4:** Pairwise comparison of MP% across follow-ups. t-Values and p-values represent pairwise comparisons between time intervals using Bonferroni correction for multiple testing, negative t-values indicate improvement (increase) in masticatory performance over time. *p-Value <0.05 denotes statistical significance. MP% = masticatory performance percentage based on almond particle size distribution [10].

Groups	3rd month vs. 6th month		3rd month vs. 12th month		6th month vs. 12th month	
	t-Value	p-Value	t-Value	p-Value	t-Value	p-Value
Single-piece implants (n = 30)	-4.92	0.001*	-5.40	0.001*	-8.16	0.001*
Conventional FPDs (n = 30)	-3.88	0.001*	-3.74	0.001*	-6.28	0.001*

## Discussion

The present study demonstrated that single-piece implant-supported crowns provided superior MP compared with conventional three-unit FPDs for replacing a single posterior tooth, with significantly higher mean MP values at three, six, and 12 months. Both groups exhibited progressive improvements over time; however, the implant group showed a more pronounced enhancement. These findings underscore the functional advantages of osseointegrated implants in restoring masticatory efficiency, aligned with the study's hypothesis that bone-anchored restorations outperform tooth-supported prostheses [11].

Our results corroborate those of previous research, highlighting the superiority of implant-based restorations in masticatory function. For instance, a systematic review by Fueki et al. [12] on the impact of implant-supported dentures on MP concluded that mandibular implant overdentures significantly enhance comminution efficiency compared with conventional complete dentures, particularly in edentulous patients with resorbed ridges. Although their focus was on overdentures, the underlying principle of rigid osseointegration for improving particle breakdown applied to our single-tooth scenario. Similarly, in a prospective study evaluating masticatory efficacy after implant rehabilitation, Montoya-Carralero et al. [11] reported reduced variance of hue in two-color gum tests and improved patient-perceived chewing ability with implant-supported prostheses, attributing gains to better occlusal force distribution and muscle symmetry, factors absent in FPDs due to PDL mediation. In contrast to FPDs, where occlusal loads are transmitted through abutment teeth and are limited by protective reflexes [13], implants bypass the PDL, allowing greater force application without inhibition [3]. This mechanistic difference likely explains the 25-26% higher MP% in our implant group across the follow-ups.

In contrast, a comprehensive analysis by Kumar et al. [7] assessing masticatory efficiency in prostheses supported by implants, as opposed to those supported by natural teeth, indicated that there were no statistically significant differences in MP and efficiency for singular teeth substituted with implants or FPDs. Similar results have been reported by Altayyar et al. [14]. Zhang et al. [15] reported that the mean ± standard deviation of the relative occlusal force for implant-supported crowns in the light occlusion cohort was inferior to that in the normal occlusion cohort. Although our study focused on MP rather than bite force, the functional correlation is evident; higher sustainable forces in implants facilitate better food comminution, as seen in our almond sieving outcomes. Discrepancies in the literature, such as no significant difference in MP between fixed-implant partial dentures and conventional removable partials in Kennedy Class I/II cases [7,14], may stem from multi-unit designs or varied test foods, whereas our single-tooth natural food protocol highlights the advantages of simplified protocols.

The temporal progression in both groups reflected neuromuscular adaptation and occlusal refinement. In the implant group, significant pairwise improvements indicated rapid integration and sensory feedback optimization, consistent with studies on implant loading protocols [16]. FPDs also improved, likely due to abutment stabilization and patient habituation, but plateaued earlier, possibly due to PDL fatigue or micromovements in pontics. These trends align with longitudinal data from Fontijn-Tekamp et al. [17], who observed gradual masticatory gains in implant overdenture users over one year, emphasizing the time-dependent cortical plasticity in chewing patterns.

Clinically, these results suggest that single-piece implants are the preferred modality for single posterior tooth replacement, especially in patients who prioritize function. Enhanced masticatory efficiency translates to better nutrient intake, reduced gastrointestinal strain, and improved quality of life [18].

This study has certain limitations that should be considered while interpreting the results. First, the study included a relatively limited sample size and was conducted at a single center, which may restrict the generalizability of the findings. Second, the study design did not include a neutral control group or baseline assessment of masticatory efficiency prior to prosthetic rehabilitation; therefore, absolute functional improvement following treatment could not be quantified. Third, although strict inclusion criteria were applied to standardize abutment conditions in the FPD group, factors such as degree of tooth preparation, taper, and individual variations in periodontal proprioception may still have influenced MP. Additionally, the follow-up period of 12 months reflects short-term functional adaptation and does not provide insight into long-term biomechanical or biological outcomes. Future randomized, multicenter studies with larger samples, baseline measurements, and extended follow-up are warranted to validate and expand upon these findings.

## Conclusions

Within the limitations of this prospective clinical study, single-piece implant-supported crowns demonstrated higher masticatory efficiency and a greater magnitude of functional improvement compared to conventional three-unit FPDs over a 12-month follow-up period. Both treatment modalities showed significant time-dependent improvement, indicating functional adaptation irrespective of the prosthetic design. While the results suggest a potential functional advantage of single-piece implants for single posterior tooth replacement, these findings should be interpreted cautiously in view of existing conflicting evidence. Further well-designed randomized controlled trials incorporating baseline functional assessments and longer follow-up periods are required before definitive clinical recommendations can be made.
